# Inborn Errors of Immunity With Fetal or Perinatal Clinical Manifestations

**DOI:** 10.3389/fped.2022.891343

**Published:** 2022-05-06

**Authors:** Magda Carneiro-Sampaio, Adriana Almeida de Jesus, Silvia Yumi Bando, Carlos Alberto Moreira-Filho

**Affiliations:** ^1^Department of Pediatrics, Faculdade de Medicina, Universidade de São Paulo, Sao-Paulo, Brazil; ^2^Translational Autoinflammatory Disease Section, National Institute of Allergy and Infectious Diseases, National Institutes of Health, Bethesda, MD, United States

**Keywords:** inborn errors of immunity, primary immunodeficiencies, hydrops fetalis, intrauterine growth retardation, familial hemophagocytic lymphohistiocytosis, IPEX, Type 1 interferonopathies, Omenn syndrome

## Abstract

In this article we revised the literature on Inborn Errors of Immunity (IEI) keeping our focus on those diseases presenting with intrauterine or perinatal clinical manifestations. We opted to describe our findings according to the IEI categories established by the International Union of Immunological Societies, predominantly addressing the immunological features of each condition or group of diseases. The main finding is that such precocious manifestations are largely concentrated in the group of primary immune regulatory disorders (PIRDs) and not in the group of classical immunodeficiencies. The IEI categories with higher number of immunological manifestations *in utero* or in perinatal period are: (i) diseases of immune dysregulation (HLH, IPEX and other Tregopathies, autosomal recessive ALPS with complete lack of FAS protein expression) and (ii) autoinflammatory diseases (NOMID/CINCA, DIRA and some interferonopathies, such as Aicardi-Goutières syndrome, AGS, and USP18 deficiency). Regarding the other IEI categories, some patients with Omenn syndrome (an atypical form of SCID), and a few X-linked CGD patients present with clinical manifestations at birth associated to immune dysregulation. The most frequent clinical features were hydrops fetalis, intrauterine growth retardation leading to fetal loss, stillbirths, and prematurity, as in HLH and IPEX. Additionally, pseudo-TORCH syndrome was observed in AGS and in USP18 deficiency. The main goal of our review was to contribute to increasing the medical awareness of IEI with intrauterine and perinatal onset, which has obvious implications for diagnosis, treatment, and genetic counseling.

## Introduction

Inborn Errors of Immunity (IEI), previously and still called Primary Immunodeficiency Diseases, represent a large and ever-growing group of rare genetic diseases with predominantly monogenic etiologies. Increased susceptibility to infections has been the hallmark clinical feature of these diseases, but other clinical phenotypes have increasingly been associated with IEIs, such as autoimmune and autoinflammatory diseases, severe allergies and benign lymphoproliferative disorders; and some IEIs are associated with development of malignant diseases (1, 2). The latest IUIS (International Union of Immunology Societies) classification of IEI issued in 2019 comprises 430 distinct genetic defects (3), and just 1 year after this publication an interim update added 26 new monogenic IEI (4).

The majority of IEI present early in life, and a significant proportion of them, particularly the most severe ones, manifest in the first months of life, some even in the neonatal period (5–10). Nevertheless, a few reports on intrauterine manifestations of IEI have been published, frequently represented by cases of hydrops fetalis in individuals in whom an IEI-related mutation was later identified in the family (7, 9, 11–17). Commonly, infectious manifestations were the first and dominant features described in IEI patients but have not been reported in intrauterine life. The interest on this topic has emerged more recently, with the recognition of primary immune regulatory disorders (PIRD) where fetal and perinatal involvement have been reported in some cases (9, 15, 16).

In the present article, we searched the literature for IEI presenting with intrauterine and/or perinatal clinical manifestations, aiming at drawing attention to the diagnosis of these conditions. We opted to describe our findings according to the IEI categories established in the IUIS, predominately addressing the immunological manifestations of each condition or group of diseases.

## Immunodeficiencies Affecting Cellular and Humoral Immunity

Severe combined immunodeficiency (SCID) is the prototype of this category, and most of the affected infants are born healthy, after uneventful pregnancies. If not diagnosed by neonatal screening and referred for transplantation, SCID patients usually present with severe or persistent infections, sometimes due to opportunistic agents, in the first months of life, associated to intractable diarrhea and failure to thrive (6, 10, 18). On the other hand, Omenn syndrome (OS), an atypical form of SCID with severe autoimmunity features, has been reported at or shortly after birth (19). In reviewing large series of infants with *RAG1* and *RAG2* mutations—the most frequent genetic defects underlying OS—there are reports of cases identified at birth or in the first week of life (20–22). However, no additional clinical details about these newborns were included in these publications, which were mainly focused on the genetic findings. There are also descriptions of generalized erythroderma at birth evolving to the typical OS skin rash lesions in few days (23–26).

**Figure 1:**
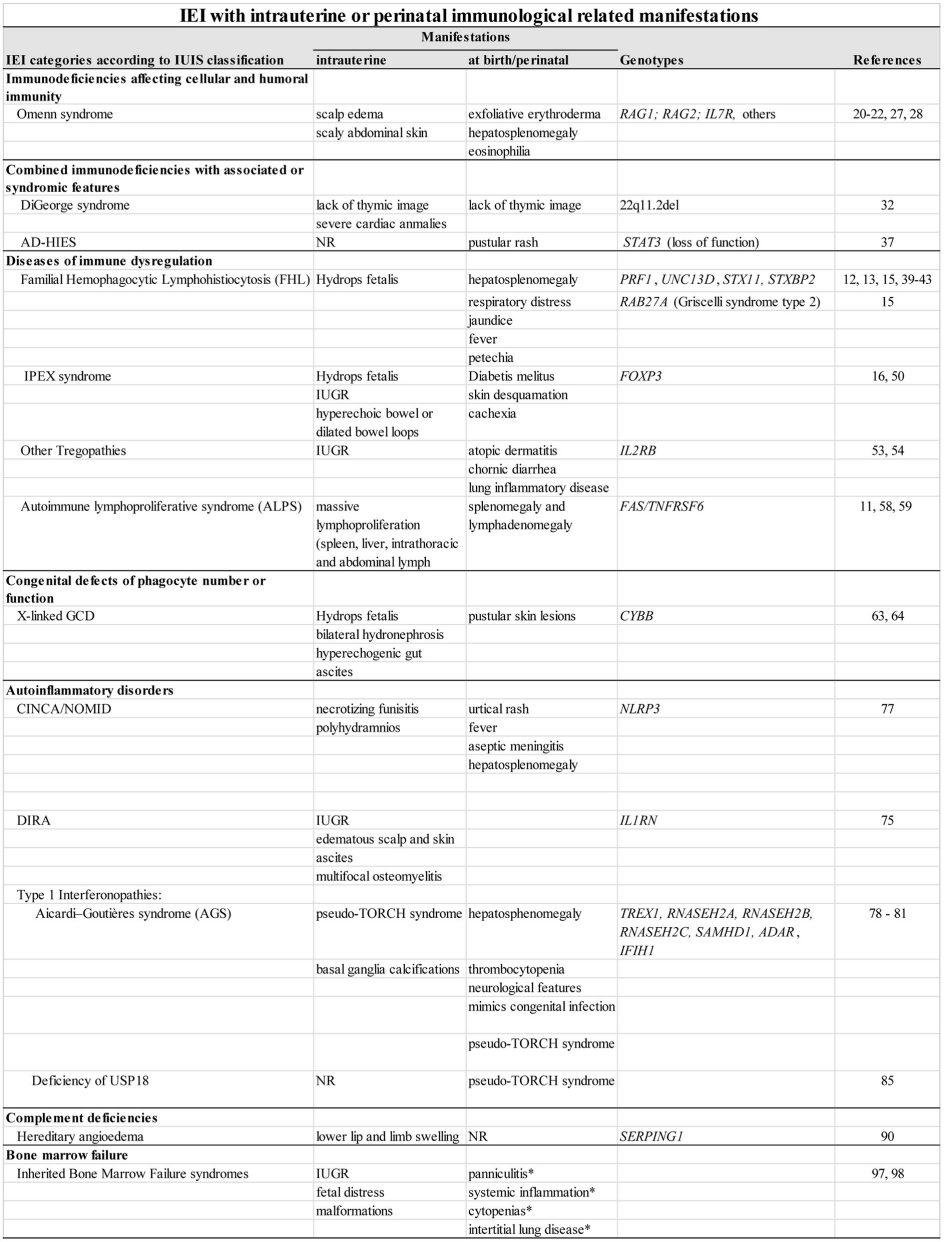
IEI with intrauterine or perinatal immunological related manifestations. NR, not yet reported; IUGR, intrauterine growth restriction; *observed in SAMD9L-associated autoinflammatory disease only.

There are a few case reports of infants presenting at birth with the characteristic features of OS (Figure 1). One of these patients, born in 2013 at a University of São Paulo hospital (HC-FMUSP), was a 28-week gestational-age female baby who had a homozygous mutation in *IL7R* (p.C118Y) (27). In addition to generalized exfoliative erythroderma, she presented, in the 1st day of life, with hepatosplenomegaly, enlarged lymph nodes, severe eosinophilia (10,335 cells/mm^3^), neutrophilia (5,625 cells/mm^3^), 2,925 lymphocytes/mm^3^, being CD3+ 684 cells/mm^3^, CD4+ 345 cells/mm^3^, CD8+ 8 cells/mm^3^, no naïve T cells, no CD127 expression, CD4+CD25+Foxp3+ 2.3%, CD19+ 641 cells/ mm^3^, IgG 468 mg/dL, IgM 45 mg/dL, IgE 3,310 UI/mL. She developed severe respiratory distress and died in the 2nd day of life. Autopsy revealed dense eosinophilic infiltrates in several organs, including pancarditis, which may have contributed to her death. Despite denying consanguinity, both parents were carriers of the same pathogenic variant. More recently, a similar case was described in which the diagnostic suspicion of ichthyosis was raised by a prenatal ultrasound that showed a fetus with significant scalp edema, echogenic amniotic fluid, and scaly abdominal skin at 31 weeks of gestational age (28). A *RAG2* mutation (c.829dupT) was identified, but, unfortunately, the infant died on the 18th day of life due to sepsis, and autopsy histological features were compatible with an OS diagnosis.

## Combined Immunodeficiencies with Associated or Syndromic Features

This is a remarkably heterogeneous category: it is the second most common IEI category identified in patient series and encompasses the second highest number of diseases. The clinical diagnostic suspicion of many of these conditions can be raised early in life usually due to the associated clinical features and not to the immunological manifestations (2, 7, 10, 29, 30). Some examples are briefly described in the following paragraphs.

The Wiskott-Aldrich syndrome is an X-linked disorder caused by mutations in the *WAS* gene, and characterized by microplatelet thrombocytopenia, eczema, recurrent infections, and an increased risk of autoimmunity diseases, lymphomas, and leukemias (31). The earliest manifestations, often present at birth, are petechiae and bruises, due to low numbers of abnormal platelets (9). Bleeding umbilical stump or excessive bleeding following circumcision and bloody diarrhea may be among the earliest WAS features (30). Severe infections and eczema, that are immunological-associated features, usually appear a little later, but in the first months of life.

DiGeorge syndrome (DGS) is caused by 22q11.2del and severe cardiac anomalies are present in 49–83% of cases (32), conotruncal defects are usually seen on prenatal ultrasound, suggesting the diagnosis (9, 32, 33). The lack of thymic image can also draw attention to DGS diagnosis. Hypocalcemia due to hypoparathyroidism is seen in approximately one half of DGS patients, often within the first few days of life, and, together with the cardiac defects, strongly suggest a DGS diagnosis (Figure 1). DGS shares many phenotypic characteristics with CHARGE (Coloboma, Heart defect, Atresia choanae, Retarded growth and development, Genital hypoplasia, Ear anomalies/deafness) syndrome, including impaired thymic development (3, 7, 34, 35). CHARGE is associated to *CHD7* mutations in most cases, and to mutations in *SEMA3E* in some patients. Complete DiGeorge anomaly (characterized as thymic aplasia) can occur in association with CHARGE (34). In the more severe forms of DGS as well as in other congenital thymic defects, TREC levels may be low in neonatal screening (18).

Some IEIs are associated with skeletal dysplasia anomalies, such as cartilage-hair hypoplasia (due to mutations in *RMRP*), Schimke immuno-osseous dysplasia (*SMARCAL1)*, Roifman syndrome (*RNU4ATAC*), and others. The diagnosis suspicion may be raised in the neonatal period or even *in utero*, with most cases presenting with intrauterine growth retardation (IUGR) (7, 9, 33, 36). Microcephaly with bird-like facial dysmorphia may be observed at birth in cases of DNA Ligase IV deficiency (*LIG4)*, Nijmegen breakage syndrome (*NBS1*), and Cernunnos-NHEJ1 deficiency *(NHEJ1)* (6, 7). In addition to a bird-like face, Bloom syndrome-affected babies (*RECQL3*) present IUGR, which may help early diagnosis suspicion (7, 33).

In the autosomal dominant (AD) forms of Hyper-IgE syndrome (HIES) due to loss-of-function *STAT3* mutations (formerly known as Job syndrome), a rash may be observed in the newborn period, and may even be present at birth (37). This rash is typically pustular, and on biopsy may be consistent with eosinophilic pustulosis. It may resolve or persist, evolving into an eczematoid dermatitis usually complicated by *Staphylococcus aureus* infection (38). The typical facial appearance, other dysmorphisms and skeletal abnormalities are not usually observed at birth, but later in childhood. Differently from AD-HIES, in DOCK8 deficiency the rash may not be present in the newborn period, but often becomes apparent later with other manifestations of this Hyper-IgE syndrome (38).

## Predominantly Antibody Deficiencies

This IEI category encompassed 39 diseases in the latest IUIS classification, which are the most frequently identified defects in IEI patient series worldwide (29). Antibody deficiencies have not been diagnosed during neonatal period, but from the second semester onwards when placental antibodies disappeared, or even later, as in the heterogeneous group of Common Variable Immunodeficiencies (CVID) (2, 3, 39). In studying 1,008 patients with well-characterized primary immunodeficiencies (PIDs) followed at our Hospital, only 17% of the cases identified under 2 years of age had “Predominantly Antibody Deficiencies,” and all of those had X-linked Agammaglobulinemia or Transient Hypogammaglobulinemia of Infancy (5). In contrast, most of the patients diagnosed in older ages were included in this category, reaching 85% of the cases among patients whose PID was identified after the third decade of life.

## Diseases of Immune Dysregulation

In this fast-growing category, the first descriptions of IEI with intrauterine manifestations were represented by fetuses with hydrops and characterized as presenting HLH (12, 13, 40–42) (Figure 1). In 2014 our group identified for the first-time intrauterine IPEX (Immunodysregulation Polyendocrinopathy Enteropathy X-linked syndrome) in two unrelated Brazilian families with multigenerational history of miscarriages of male fetuses due to hydrops (14). Prenatal manifestations were observed in a few fetuses with a genomic homozygous deletion of FAS-encoding-gene, the most frequent underlying defect in Autoimmune Lymphoproliferative Syndrome (ALPS) (11).

### Familial Hemophagocytic Lymphohistiocytosis

Primary or familial hemophagocytic lymphohistiocytosis (FHL) was one of the first IEI where intrauterine onset has been described in cases of non-immune hydrops fetalis, and, in more recent reports, hemophagocytic lymphohistiocytosis (HLH)-associated gene mutations were identified (12, 13, 15, 40–43). FLH is characterized by functional defects of cytotoxic T lymphocytes and NK cells, and associated with mutations in various genes, being *PRF1* (FHL2), *UNC13D* (FHL3), *STX11* (FHL4), and *STXBP2* (FHL5) the most frequently identified so far (3). Patients with some syndromes with hypopigmentation and defective lymphocyte granule-mediated cytotoxicity, such as Griselli syndrome type 2 (due to *RAB27A* mutations), Chédiak-Higashi syndrome (*LYST* mutations) and Hermansky-Pudlak syndrome type 2 (*AP3B1* mutations) develop HLH, sometimes in early life (44–47). The X-linked lymphoproliferative diseases (XLP) caused by pathogenic variants of *SH2D1A* (XLP1) and *XIAP* (XLP2) can also present with HLH (46).

FHL frequency is estimated as 1:50,000 live births (44). Usually, the affected infants are asymptomatic at birth but many of these patients presents with clinical manifestations in the first year of life and in ~10%, the onset occurs in the neonatal period (42). Among the 20 cases identified as HLH in a nationwide survey in neonates conducted in Japan between 1997 and 2007, six had manifestations at birth and five during the first week of life (48). Despite the significant number of descriptions of intrauterine- or perinatal-onset of FHL, it is not possible to estimate the frequency of these cases (15). Fetal-onset FHL is considered the most severe form of the disease and carries a high mortality, close to 95% (48, 49).

In reviewing 46 FHL patients who presented with initial symptoms in intrauterine or perinatal periods, hydrops fetalis was the most frequent manifestation (71%), being usually detected after 30 weeks of gestational age (GA), although there are cases described at the 24th week of GA, leading to stillbirths and prematurity with high mortality (12, 13, 15, 40–43). Additionally, hepatosplenomegaly (50%), respiratory distress (27%), fever (27%), jaundice (23%), and petechiae (19%) were the most frequent initial symptoms at birth or perinatal age. FHL2 (48%) was the most frequent type identified in genetically tested individuals (*n* = 27), followed by FHL3 (22%) and FHL5 (15%). Only one patient had Griscelli syndrome type 2. No cases of intrauterine or perinatal FHL4, XLP or Chédiak-Higashi syndrome were identified. In 19 individuals the genetic characterization was not performed.

### Immunodysregulation Polyendocrinopathy Enteropathy X-Linked Syndrome (IPEX)

IPEX is caused by mutations in *FOXP3* impairing the development of T regulatory cells. It is known that IPEX manifestations may arise in fetal life, and intrauterine IPEX forms certainly represent the earliest onset of autoimmune diseases in humans (16). Among the 94 reported IPEX cases with “age-of-onset” information, 46 (49%) presented with the first symptoms during the neonatal period, and 11 presented with typical features at birth or in the first 2 days of life (11.5%), drawing attention to antenatal initiation of the pathological process (50).

We recently reviewed the clinical, histopathological, and genetic findings in 21 individuals from 11 unrelated families, with nine different mutations, all described as cases of intrauterine IPEX (16). Recurrent male fetal death (multigenerational in five families) due to hydrops fetalis in the midsemester of pregnancy was the most common presentation (13/21). Noteworthy, in the affected families, there were only fetal- or perinatal-onset cases, with no affected individuals presenting with milder forms and later-onset manifestations. Most live births were preterm (5/6). Skin desquamation and IUGR were observed in part of the cases. Fetal ultrasonography showed hyperechoic bowel or dilated bowel loops in the 5 cases with available imaging data. Histopathology evaluation showed multi-visceral infiltrates with T lymphocytes and other cells, including eosinophils, the pancreas being affected in most cases (11/21), and as early as at 18 weeks of gestational age. Regarding the nine *FOXP3* mutations found in these cases, six lead to protein truncation and 3 predictably impair protein function, although no clear genotype-phenotype correlations could be drawn from these cases. Intrauterine IPEX seems to constitute a particular subgroup, certainly the most severe clinical presentation of IPEX.

We recently assisted a 6-month-old boy with IPEX presenting with persistent diarrhea with failure to thrive, who had had the diagnosis of hypothyroidism in the neonatal screening and developed a Hashimoto's disease with high titers of anti-thyroid peroxidase (TPO), anti-thyroglobulin (TGB) and anti-TSH receptor autoantibodies. The mother was healthy and did not have any thyroid diseases. This case is probably a consequence of intrauterine autoimmune thyroiditis associated to IPEX, reinforcing our hypothesis that the first target organ/tissue determines the course of intrauterine IPEX, being autoimmune hemolytic anemia the cause of hydrops (16).

### Other Monogenic Tregopathies

We also reviewed the age-of-onset data from other monogenic Tregopathies leading to IPEX-like phenotypes and found clear intrauterine onset only in part of those patients affected by defects of IL-2 receptor β chain (16, 51, 52). Among the five families so far described with *IL2RB* mutations, two had intrauterine affected individuals, being two preterm babies, and two stillbirths, all apparently with severe growth restriction, and unfortunately none survived (53, 54). Among the four cases of CD25 deficiency so far described, i.e., affecting the IL-2 receptor α chain, only one started clinical manifestation on the 6th day of life, with severe atopic dermatitis, chronic diarrhea, and lung inflammatory disease (55).

### Autoimmune Lymphoproliferative Syndrome

Autoimmune lymphoproliferative syndrome (ALPS) is a group of disorders primarily due to impaired lymphocyte apoptosis, whose typical forms are caused by defects in the FAS pathway (FAS, FASLG, and CASP10) (56). Most patients harbor pathogenic variants in the *FAS* gene inherited in an autosomal dominant fashion, and exert a dominant-negative effect in the FAS pathway. Characteristic clinical findings include benign, chronic lymphadenopathy and splenomegaly, autoimmune cytopenias, and a high risk for the development of lymphoma. The classical laboratory hallmark of ALPS is the presence of circulating mature double-negative T cells (α/β receptor-carrying T cells that do not express CD4 or CD8 (57). Prenatal manifestations have been described in a few fetuses with genomic homozygous deletion of *FAS*/*TNFRSF6* (11, 58, 59). Le Deist et al. observed prenatal onset of massive lymphoproliferation, which involved the spleen, the liver, as well as intrathoracic and abdominal lymph nodes in a fetus with a homozygous mutation and a complete lack of FAS protein expression, whose siblings with heterozygous mutations presented with late-onset manifestations, thus, suggesting that severity of the disease is probably related to the degree of functional FAS deficiency (11, 56). Antenatal features were also detected in another unrelated fetus with a homozygous single nucleotide variant in the splice acceptor of intron 3 that resulted in the skipping of exon 4 and complete loss of FAS expression (58). In a pioneer description of clinical spectrum of FAS deficiency, three affected neonates presented with splenomegaly and lymphadenomegaly but not cytopenias, which were only seen in older children (59).

## Congenital Defects of Phagocyte Number or Function

The defects included in this category usually have an early disease onset, often in the neonatal period, with high susceptibility to severe bacterial and fungal infections, and encompass: (i) the genetically heterogeneous group of congenital neutropenias, (ii) defects of leukocyte motility, (iii) defects of respiratory burst, in addition to a few other defects, including GATA2 deficiency (3, 60). Newborns with leukocyte adhesion defects show omphalitis and a characteristic delayed separation of the umbilical cord (above 30 days of life), which is considered as an early warning sign of IEI (61, 62). In this category we were able to identify just a few cases of Chronic Granulomatous Disease (CGD) with *in utero* manifestations (63, 64).

Among the functional defects, CGD—caused by mutations in the NADPH oxidase pathway genes, leading to impaired superoxide production and defective microbial killing—is certainly the most studied disease. Besides the high susceptibility to infections, dysregulation of inflammatory responses is often present and may lead to granulomatous lesions, mostly affecting the gastrointestinal and urinary tracts (65). Regarding intrauterine manifestations, there are only a few reports in X-linked CGD cases due to *CYBB* mutations, which represent around 70% of all CGD cases (63, 64) (Figure 1). In the first case description, hydrops with pericardial effusion was detected in a 33-week gestational-age fetus who, after birth, presented with widespread pustular skin lesions, from which no bacterial or viral agents were isolated (63). The baby was diagnosed because the mother was identified as an asymptomatic carrier of X-linked CGD. The cause of hydrops was not elucidated, and the follow-up of this baby was not reported. Another publication describes a case in which an ultrasonography in the third trimester of pregnancy revealed polyhydramnios, bilateral hydronephrosis, hyperechogenic gut, and ascites; post-natal investigation concluded that he had inflammatory involvement of the gastrointestinal and urinary tracts (64). An older brother, also affected by X-linked CGD, presented with severe feeding issues and failure to thrive. Biopsies of esophagus and stomach performed at 6-months-old revealed eosinophilic infiltration of the mucosa and submucosa. Therefore, in this latter family, severe early-onset CGD inflammatory manifestations were observed—*in utero* in the second sibling—who might have had antenatal obstructive lesion of gut and bladder.

## Defects in Intrinsic and Innate Immunity

This category harbors the highest number of IEI comprising a total of 59 different diseases determined by mutations in 71 genes, according to an update of the latest IUIS classification (2, 3). All affected patients present with high susceptibility to severe infections and, in general do not have manifestations of immune dysregulation. This category mostly encompasses cytokine production defects, cell-signaling disturbances, and defective expression of many types of cell receptors and molecular sensors in several cell lineages (66, 67). The classification of the different disease subgroups included in this category was largely established according to distinct susceptibilities to determined etiological agents, or to a narrow range of microbes, i.e., predominantly to mycobacterial diseases, to viruses in general or to particular types of viruses, to certain fungal infections, etc. (3, 67, 68).

A large group of defects in intrinsic and innate immunity arise from gene mutations affecting core molecules of the TLR and TNFR-signaling pathway, involving the genes *NEMO, IKBA, IKBKA, IKBKB, HOIL1*, and *HOIP*, or, in mutations impairing the TLR-IL-1R pathway, like those in *IRAK4, MYD88, TIRAP*, and *IRAK1* (68, 69). Some of such diseases manifest in infancy or in early childhood. The autosomal recessive IKKα deficiency (*IKBKA*) and the X-recessive NEMO deficiency, both caused by hypomorphic mutations affecting the canonical NFκB pathway, show a spectrum of phenotypes ranging from fetal death, in complete IKKα deficiency (70), to severe susceptibility to infections and ectodermal dysplasia (EDA-ID phenotype) in newborns (68, 71). Another good example is the complete STAT1deficiency, a severe condition that leads to elevated susceptibility to mycobacteria and viruses and high mortality (72). In analyzing 32 patients, 29 with information on the age of onset, Le Voyeur et al. (2021) showed that 19 (66%) had their manifestations within the first 3 months of life. In this patient series, there is a mention to a female baby whose illness started in the first days of life (age of onset – 0.1 month), without further details or description elsewhere.

## Autoinflammatory Disorders

The autoinflammatory disorders are genetically defined multisystem immunodysregulatory disorders caused by mutations in genes that encode molecules of the innate immune system (73) (Figure 1). The IEI classification categorized the autoinflammatory disorders as those with (i) recurrent inflammation/fever, (ii) systemic inflammation and urticarial rash, (iii) sterile inflammation of bone and/or skin, (iv) Type I interferonopathies, and (v) miscellaneous/others. Most autoinflammatory disorders present in childhood and neonatal onset is predominantly observed in patients with: the sterile osteomyelitis syndrome, DIRA; the most severe form of the cryopyrin associated periodic syndromes (CAPS), NOMID/CINCA; and the Type I interferonopathies, such as AGS, SAVI and USP18 deficiency.

### Deficiency of IL-1 Receptor Antagonist (DIRA)

DIRA is caused by loss-of-function mutations in *IL1RN*, encoding the IL-1 receptor antagonist (IL-1Ra), that lead to increased signaling through the IL-1 receptor. This ultra-rare IL-1 mediated autoinflammatory disease has a very early onset and the majority of the 30 cases so far reported presented with the first clinical manifestations between birth and the 3rd week of life (74).

There is one case report of DIRA that showed evidence of an intrauterine disease onset in two siblings born to consanguineous parents who had a homozygous *IL1RN* nonsense mutation (c.355C>T, p.Gln119^*^) (75). One child presented with intrauterine demise at 27 weeks of gestational age. A routine ultrasound at 23 weeks of gestational age showed IUGR and edematous areas of the scalp and skin, minimal ascites, short ribs, narrow thorax, and polyhydramnios. This fetus died at 27 weeks of gestational age and post-mortem x-ray indicated osteomyelitis. Histopathological examination showed abscesses with dense neutrophil infiltrates and extensive tissue destruction in thymus, adrenal gland, and myocardium. The second child was born at 31 weeks of gestational age and presented with pustular lesions on the scalp and extremities, multifocal osteomyelitis, and raised CRP levels in the first week of life. At the age of 3 months, the patient presented with worsening respiratory distress and succumbed to a CMV infection with macrophage activation syndrome at the age of 9 months (75).

### Neonatal-Onset Multisystem Inflammatory Disease (NOMID)/Chronic, Infantile, Neurological, Cutaneous and Articular (CINCA) Syndrome

NOMID is the most severe clinical phenotype of a disease spectrum caused by gain-of-function mutations in *NLRP3*, called the Cryopyrin Associated Periodic Syndromes (CAPS). Patients with NOMID usually present with an urticarial rash, continuous fever and aseptic meningitis in the first hours of life and, overtime, develop sensorineural hearing loss and bony overgrowth (73).

In 1987, Prieur et al. described the clinical features of 30 patients with the newly reported syndrome CINCA/NOMID. In that study, data on perinatal events were available in 20 of the 30 cases. Eleven of these 20 patients were premature and three of them had birth weight below the 10th percentile. Two cases of polyhydramnios were reported, and umbilical cord anomaly was observed in five patients, three of them with omphalocele, one with thickened and gelatinous umbilical cord and one with omphalitis. Histological study of the placenta in one case showed thickened vessel walls, with thrombosis and calcifications of basal and intrachorial membranes. Microcalcifications were also seen in the cord vessel walls with numerous polymorphonuclear cells infiltrating the umbilical cord (76).

Recently, Yokoi et al. (77) reported a preterm NOMID female patient that presented with necrotizing funisitis as an intrauterine manifestation. The patient was born at 33 weeks and 5 days of gestational age due to polyhydramnios, spontaneous preterm labor and increased maternal CRP. At birth, the patient presented with severe hepatosplenomegaly and mild ventriculomegaly and, a few hours after birth, she developed a generalized urticarial rash. Laboratory exams showed elevated CRP (64 mg/L), leukocytosis (WBC 34,650/μL), and an aseptic neutrophilic meningitis. The patient was found to have a pathogenic mutation in *NLRP3* (c.1698C>G, p.Phe566Leu) and was started on an IL-1 inhibitor at the age of 2.5 months, with complete resolution of the clinical manifestations by the age of 4 months (77). Edema and yellow discoloration were noticed on the umbilical cord; the placenta was friable and weighted >90th percentile for the gestational age. Histopathological examination showed necrotizing funisitis, namely inflammation of the umbilical cord, which was limited to one umbilical artery, associated with focal neutrophilic and lymphocytic villitis (77).

### Type 1 Interferonopathies

Aicardi–Goutières syndrome (AGS) represents the prototype of this group of monogenic diseases characterized by a constitutive upregulation of Type 1 IFN (78, 79). AGS may be caused by mutations in any of these genes: *TREX1, RNASEH2A, RNASEH2B, RNASEH2C, SAMHD1, ADAR, IFIH1, LSM11*, and *RNU7-1*. These findings lead to the identification of seven subgroups of the disease (AGS 1-9) (78, 80, 81). AGS is a progressive encephalopathy with early onset, sometimes in the intrauterine period, characterized by basal ganglia calcifications, white matter destruction, brain atrophy with chronic cerebrospinal fluid (CSF) lymphocytosis and high Type 1 interferon levels in the CSF without evidence of congenital infections, and was originally defined as a pseudo-TORCH (toxoplasmosis, rubella, cytomegalovirus, and herpes) syndrome (80).

Among the 374 patients with AGS evaluated in the largest multicenter study on this condition so far published, 74 patients (22.8%) demonstrated signs of *in utero* disease-onset (82). Thirty-seven infants (25 *TREX1*; two *RNASEH2A*; one *RNASEH2B*; three *RNASEH2C*; three SAMHD1; one *ADAR*; two *IFIH1*) presented at birth with a clinical picture mimicking a congenital infection comprising abnormal neurological signs (poor feeding, irritability, abnormal tone, abnormal movements and seizures), hepatosplenomegaly and thrombocytopenia, and other 37 (13 *TREX1*; one *RNASEH2A*; nine *RNASEH2B*; seven *RNASEH2C*; six *SAMHD1*; one *ADAR*) showed neurological features at birth without obvious systemic involvement (82).

AGS pathogenesis remains largely to be characterized. However, regarding the neurological involvement, Type 1 IFN has been implicated in regulating microglia—the local component of the innate immune system—during development, when it has essential homeostatic roles in sculpting neural circuitry and coordinating diverse neurodevelopmental processes (80, 83, 84).

Besides AGS, other Type 1 interferonopathies should be of particular interest regarding their impact in early life, as the series of the new diseases expand and their phenotypes become better characterized (80). An example is the Deficiency of USP18, that was also described as pseudo-TORCH syndrome in five individuals from two families, being all of them affected, three still detected *in utero* and two at birth, without survivors (85). Ubiquitin-specific peptidase 18 (USP18) is a key negative regulator of type I IFN signaling, and the disease results from loss-of-function recessive mutations.

Most cases of STING-associated vasculopathy with onset in infancy (SAVI) have their first manifestations in early life (86). SAVI is associated with gain-of-function mutations in *STING1* (stimulator of interferon genes) gene, leading to increased expression of interferon-stimulated genes and Type I interferon proteins (IFNβ and IFNα). Frémond et al. (86) described a SAVI cohort of 21 cases and reviewed 40 literature cases and among the 61 cases reported, 60% had a disease onset before the age of 6 months. In a systematic review, Dai et al. (87) described 51 SAVI cases reported up to 2020, showing that 17 (33.3%) were neonates, but detailed information on the exact age-of-onset was not available even exploring the isolated descriptions.

## Complement Deficiencies

Thirty-six diseases have so far been recognized in this category, mainly with autosomal recessive inheritance, being Hereditary Angioedema (HAE) the most frequent one (1:10,000–50,000) (88, 89). The immune-related manifestations of Complement deficiencies, either bacterial infections or rheumatic diseases, usually have childhood onset (Figure 1). We were able to identify only one case of HAE with an intrauterine attack (90). A 23-year-old pregnant woman in the 38th week of gestational age and with a previous HAE diagnosis presented with a HAE episode and referred an unusual abdominal sensation; fetal ultrasonography was performed at that moment and detected fetal lower lip swelling (3 times the normal size) and limb swelling. Genetic analysis later confirmed that the male infant had type I HAE (mutation in *SERPING1*). Treatment of the mother with recombinant human C1-INH was effective for both maternal and fetal attacks, which occurred almost simultaneously. This observation may be a warning sign in the obstetric management of HAE patients since it is well-known that women are more likely to have severe HAE attacks, and they occur more often during pregnancy (91).

It is also worth to mention 3MC syndrome, an autosomal recessive complement deficiency which may exert severe impacts on fetal development and organ ontogenesis. It is caused by homozygous recessive mutations in the lectin pathway activation genes *COLEC10, COLEC11*, and *MASP1/3* (92, 93) that impede the correct migration of neuronal crest cells during embryogenesis. Affected individuals present with facial dysmorphism, cleft lip and/or palate, craniosynostosis, learning disability, and genital, limb and vesicorenal anomalies. The 3MC syndrome clearly indicates a role for complement proteins in developmental processes (92–94), which was recently confirmed by the finding that rare biallelic variants in the gene encoding the complement binding protein C1QBP may cause intrauterine disease onset with IUGR, cardiomyopathy, oligohydramnios, hydrops fetalis, and lethal preterm outcome (95).

## Bone Marrow Failure

This category was included in the latest IUIS classification of IEIs in 2019 encompassing 43 defects, and its most important diseases are: (i) Fanconi anemia (FA), due to mutations in 22 genes, almost all with AR inheritance, (ii) Dyskeratosis Congenita (DKC), caused by mutations in 14 different genes, with autosomal dominant (AD), AR and X-linked recessive inheritance, and all causing dysfunctional (short) telomeres, (iii) *SAMD9/SAMD9L* syndromes (3). Besides FA and DKC, this large group of diseases so-called “Inherited Bone Marrow Failure syndromes” (IBMFS) also includes Diamond Blackfan Anemia (DBA) and Shwachman Diamond Syndrome (SDS), and, more recently, several additional conditions were added, such as GATA2 deficiency and the *SAMD9/SAMD9L* syndromes (96).

Clinical diagnostic suspicion of a bone marrow failure disease is commonly raised in childhood, or even in early adulthood, due to persistent cytopenias associated with hypocellular bone marrow, or when there is a familial history (96). However, in fact, part of these IBMFS–affected individuals present intrauterine manifestations, such as malformations (e.g., radial or thumb anomalies), IUGR and fetal distress (97). In a large retrospective analysis, Giri et al. studied the outcomes of 575 pregnancies in 165 unaffected mothers of offspring with FA, DKC, DBA and SDS, and observed that FA-affected babies were more frequently born small for gestational age (SGA) than unaffected controls (39 vs. 4%), and had malformations (46%) with 18% with three or more anomalies; DKC offsprings had higher rates of SGA (39 vs. 8%) and fetal distress (26 vs. 3%). Nevertheless, clinical manifestations due to immunodeficiency—usually associated to cytopenias—appear later in life (96). In addition to an ataxia-pancytopenia syndrome (APS), *SAMD9L* mutations can cause an early-onset severe autoinflammatory disorder called SAMD9L-associated autoinflammatory disease (SAAD). Differently from APS, SAAD is caused by frameshift mutations that affect a specific region on the SAMD9L protein and it is clinically characterized by a neonatal onset of systemic inflammation, neutrophilic panniculitis, variable cytopenias, and interstitial lung disease (98) (Figure 1).

## Discussion

Intrauterine and perinatal manifestations have predominantly been observed in the so-called primary immune regulatory disorders (PIRDs), contrasting with the classical immune deficiencies, where infection is the main clinical feature, certainly due to the anti-infectious protection offered by the intrauterine environment and placental transfer of antibodies. Taken as a whole, IEI with fetal and/or perinatal manifestations revealed that immune dysregulation affecting various cells types and immune pathways may lead to very early onset of: (i) autoimmune manifestations, as seen in IPEX and in OS; (ii) autoinflammatory aggression, caused by inflammasome dysregulation, as in CINCA/NOMID and DIRA, or by upregulated production of type I IFN, as in AGS and in USP18 deficiency; (iii) hyperinflammation derived from lymphocyte cytotoxicity defects, as in FHL, due to various gene mutations, (iv) benign lymphoproliferation, as observed in a few fetuses with homozygous *FAS* mutations.

It is not possible to estimate the frequency of intrauterine-onset cases among those affected by these rare diseases. Considering the category of autoinflammatory diseases, the percentages of fetal- and perinatal-onset cases are the highest ones among the IEIs here approached: (i) most cases of NOMID/CINCA and DIRA already had manifestations at birth and (ii) approximately a quarter of AGS cases were clearly affected at birth, as well as all the few USP18 deficiency cases so far described, thus revealing that pathological processes already had begun during the gestational period (82, 85). In IPEX, it could be estimated that at least 10% of the affected individuals present clinical signs at birth (50). Since the intrauterine outcome is usually a midsemester miscarriage, part of these cases may not have been identified yet (16). Even for FHL—certainly the least rare among the diseases addressed here—it is not possible so far to estimate the percentage of cases with intrauterine onset (15). It is worth calling attention to the fact that all these diseases were described only recently, and that their very early onset has been little explored to date. On the other hand, antenatal manifestations in X-linked CGD, ALPS, HAE appear to be, in fact, very rare, with few descriptions so far in these not-so-infrequent and well-known IEIs, but they should be investigated, especially when there is a positive family history.

Except for AGS, USP18 deficiency, and probably other Type 1 interferonopathies, in which most precocious manifestations mimic a TORCH syndrome, in the IEI diseases addressed here (HLH, IPEX, defects of IL-2 receptor β chain, X-linked CGD, NOMID/CINCA), the intrauterine manifestations are quite similar, presenting mainly as hydrops and IUGR, which leads to fetal losses, stillbirths, and prematurity (15, 16). Umbilical cord and placental anomalies have been described only in NOMID/CINCA (76, 77). Skin manifestations at birth may be extremely relevant for an early clinical diagnostic suspicion of OS, AD-HIES, CINCA/NOMID, and even of DIRA and CGD.

We would like to make an additional comment on the IPEX case that we recently assisted, and which apparently began because of autoimmune congenital hypothyroidism i.e., as a Hashimoto's disease. We could not find a similar description in the literature, but medical awareness should be raised considering that currently all congenital hypothyroidism cases are already detected by neonatal screening tests in many countries and, thus, *FOXP3* should be included among the genes to be investigated in babies with confirmed hypothyroidism (99). In fact, hypothyroidism is the second most frequent autoimmune endocrinopathy in IPEX, second only to diabetes mellitus, and lymphocytic infiltrate in the thyroid has already been observed in some cases of intrauterine IPEX (16, 100).

Finally, an important issue that needs to be addressed is the supposed “immaturity” of the fetal immune system. The developing human fetus is extensively exposed to foreign proteins and cells transferred from the mother through the placenta, but the immune response to these antigens is attenuated by fetal dendritic cells that are fully functional by 13 weeks of gestation. Rather than helping to mount a vigorous immune response, these dendritic cells activate regulatory T cells (Tregs) and help to keep the homeostasis of fetal immune system. Fetal dendric cells accomplish this task by strongly promoting Tregs induction and, simultaneously, by controlling T cell TNF-α levels along gestation through arginase-2 activity (101). Interestingly, it was shown that human fetal and adult hematopoietic stem cells give origin to distinct T cell lineages, the fetal T cell lineage being biased toward immune tolerance (102, 103).

The rearrangement of T and B antigen receptors in thymus and bone marrow begins at 8 weeks of gestation. The mechanisms responsible for the diversity of the antigen receptor repertoire are functional in the fetus by the end of the second trimester—although the composition of this repertoire differs from that of adults due to the preferential use of certain V-D-J gene segments—and by that time T cells are already capable of clonal expansion in response to antigens (103, 104). Recently, Mishra et al. (105) by analyzing fetal tissues and placenta collected in the 2nd trimester of gestation were able to show the activation of memory T cells in fetal mesenteric lymph nodes in response to very sparse microbial presence (detected by 16SrRNA gene sequencing), thus giving additional evidence of immune competence before birth. Therefore, the fetal immune system is not immature, it is unique. A better understanding of such uniqueness is mandatory to improve the early diagnosis of IEI, as well as the pathogenesis of recurrent abortions and autoimmune disorders.

In conclusion, despite of IEI manifestations in early life, little attention has been drawn to the intrauterine manifestations of this large and heterogeneous group of diseases and, as far as we know, this article represents the first comprehensive review on this subject. Therefore, our main objective in this article was to increase the medical awareness on early IEI diagnosis, which could be crucial for the implementation of adequate therapeutic measures aiming to the survival of the affected fetuses and infants (106, 107). Neonatal screening has been an effective measure for early detection of IEI with severe T-cell lymphopenia. Nevertheless, a TREC-based screening does not detect most of the diseases addressed here. Thus, the expansion of neonatal screening seems to be essential and should include next generation sequencing techniques, which show some promising results already available applying whole genome or exome sequencing as the first-tier test (108, 109). Regarding the treatment perspectives, it is noteworthy that many IEI monogenic diseases appear to be good candidates for gene therapy, e.g., X-linked SCID and X-linked GCD, ADA-SCID and Wiskott-Aldrich Syndrome, and several clinical trials have already been accomplished (107). The transition from engineered viral vectors to gene editing approaches, coupled with advances in hematopoietic stem cell transplantation, may bring new perspectives to this field of therapeutic research (17, 107), Furthermore, the possibility of *in utero* gene therapy is already on our horizon (17).

## Author Contributions

All authors listed have made a substantial, direct, and intellectual contribution to the work and approved it for publication.

## Funding

This work was supported by Fundação de Amparo à Pesquisa do Estado de São Paulo (FAPESP) grant 2014/50489-9 to MC-S. Funding was provided in part by the Intramural Research Program of NIAID, NIH (for A.A.D.J).

## Conflict of Interest

The authors declare that the research was conducted in the absence of any commercial or financial relationships that could be construed as a potential conflict of interest.

## Publisher's Note

All claims expressed in this article are solely those of the authors and do not necessarily represent those of their affiliated organizations, or those of the publisher, the editors and the reviewers. Any product that may be evaluated in this article, or claim that may be made by its manufacturer, is not guaranteed or endorsed by the publisher.
